# Age and Sex Influence the Hippocampal Response and Recovery Following Sepsis

**DOI:** 10.1007/s12035-019-01681-y

**Published:** 2019-07-05

**Authors:** Jolie Barter, Ashok Kumar, Julie A. Stortz, McKenzie Hollen, Dina Nacionales, Philip A. Efron, Lyle L. Moldawer, Thomas C. Foster

**Affiliations:** 1 Department of Neuroscience, McKnight Brain Institute, University of Florida, PO Box 100244, Gainesville, FL 32610-0244 USA; 2 Department of Surgery, University of Florida, Gainesville, FL 32611 USA; 3 Genetics and Genomics Program, University of Florida, Gainesville, FL 32611 USA

**Keywords:** Sepsis, Aging, Mice, Cecal ligation and puncture, Hippocampus, Transcriptome

## Abstract

Although in-hospital mortality rates for sepsis have decreased, survivors often experience lasting physical and cognitive deficits. Moreover, older adults are more vulnerable to long-term complications associated with sepsis. We employed a murine model to examine the influence of age and sex on the brain’s response and recovery following sepsis. Young (~ 4 months) and old (~ 20 months) mice (C57BL/6) of both sexes underwent cecal ligation and puncture (CLP) with restraint stress. The hippocampal transcriptome was examined in age- and sex-matched controls at 1 and 4 days post-CLP. In general, immune- and stress-related genes increased, while neuronal, synaptic, and glial genes decreased 1 day after CLP-induced sepsis. However, specific age and sex differences were observed for the initial responsiveness to sepsis as well as the rate of recovery examined on day 4. Young females exhibited a muted transcriptional response relative to young males and old females. Old females exhibited a robust shift in gene transcription on day 1, and while most genes recovered, genes linked to neurogenesis and myelination continued to be downregulated by day 4. In contrast, old males exhibited a more delayed or prolonged response to sepsis, such that neuronal and synaptic genes continued to decrease while immune response genes continued to increase on day 4. These results suggest that aging is associated with delayed recovery from sepsis, which is particularly evident in males.

**Electronic supplementary material:**

The online version of this article (10.1007/s12035-019-01681-y) contains supplementary material, which is available to authorized users.

## Introduction

Earlier recognition and treatment of sepsis has led to improved short-term survival; however, survivors of sepsis face complicated clinical trajectories, including an increased risk of cognitive impairment [1–6]. Sex is a major factor contributing to variability in the response to sepsis since many studies in humans and rodents have demonstrated decreased mortality following sepsis for females [7–12]. While the mechanism behind this sexual dimorphism remains uncertain, one explanation is that sex steroids affect the immune response to sepsis [7, 8, 10, 13]. Age is another major influential factor in the outcome of sepsis since the incidence of sepsis and the associated complications increase with advancing age [14–17]. The complications may result from dysregulation of the immune system during aging termed immunosenescence, resulting in a delayed, attenuated, and prolonged response [1, 3, 18–20].

One of the key problems facing sepsis survivors is acute and chronic cognitive deficits, particularly impaired hippocampal-dependent memory [4, 21, 22]. Studies that examine the effects of systemic inflammation on the hippocampus indicate multiple possible mechanisms including increased microglial activation [23, 24], activation of apoptotic pathways [22, 25, 26], oxidative damage [27], mitochondrial dysfunction [28], and decreased synaptic plasticity [29–33]. These effects of sepsis may be amplified with advanced age since aging is also associated with an increase in microglial activation, oxidative damage, mitochondrial dysfunction, and altered synaptic plasticity [34–36].

In the current study, we utilized a murine abdominal sepsis model [37–39] to determine how advanced age and sex influence the hippocampal transcriptome, 1 or 4 days following induction of sepsis. Next-generation sequencing, which permits examination of thousands of genes, was employed to determine the expression profile of hippocampal genes following sepsis. The results indicated that sepsis increases transcription of immune/stress response genes and decreases the expression of genes linked to the neuron, synapse, and myelination. Furthermore, the results confirmed that sex and age significantly influence the transcriptional response and recovery following sepsis.

## Methods

### Animals

Procedures involving animals were reviewed and approved by the Institutional Animal Care and Use Committee of the University of Florida and were in accordance with guidelines established by the U.S. Public Health Service Policy on Humane Care and Use of Laboratory Animals. Young (3–5 months) and old (18–22 months) male and female (C57BL/6) mice were purchased from Jackson Laboratory (Bar Harbor, ME). Mice were cared for by the University of Florida Animal Care Services and housed in transparent cages (4 animals per cage) within specific pathogen-free facilities. The animals were provided standard rodent chow and water ad libitum for the duration of the study. Prior to initiation of the experiment, mice were acclimated to the temperature- and humidity-controlled housing room programmed for a 12-h light–dark cycle for 1 week.

To induce sepsis, young and old female (50%) and male (50%) mice underwent cecal ligation and puncture (CLP), which consisted of a midline laparotomy, ligation of the cecum (~ 1 cm from its tip), and cecal puncture with a 22-gauge needle [37–39]. Immediately after surgery, the mice received a subcutaneous injection of buprenorphine diluted in 1 ml of saline. For this murine model, antibiotics and restraint stress were employed to partly mimic the clinical situation of humans in the intensive care unit. Mice received delayed subcutaneous antibiotic injections (imipenem monohydrate; 25 mg/kg diluted in 1 ml 0.9% sodium chloride) starting 8 h after CLP and twice a day thereafter for a total of 2 doses in the mice sacrificed on postoperative day (POD) 1 and 6 doses in mice sacrificed on POD4. In addition, mice were subjected to 1 h of daily stress via cone restraint in order to mimic the stress of the intensive care unit. Mice that were sacrificed 1 day after CLP received restraint cone stress 1 h prior to sacrifice, while mice sacrificed on POD4 received restraint stress on days 2–4, including 1 h prior to sacrifice. No mortalities were observed for all animals on day 1. There were no mortalities in young mice on day 4, while old mice irrespective of sex exhibited a mortality rate of 58.8% on day 4.

### Tissue Collection

Animals were sacrificed either 1 or 4 days following sepsis. Age- and sex-matched control groups were sacrificed directly from the home cage, without receiving surgery or anesthesia. Mice were anesthetized with isoflurane (Halocarbon Laboratories, River Edge, NJ) and swiftly decapitated, and mixed arteriovenous blood was collected in a heparinized vacutainer tube. The brains were rapidly removed and the hippocampi were dissected. All brain samples were flash frozen in liquid nitrogen and were stored at − 80 °C. One whole hippocampus was used for next-generation sequencing.

### Plasma Cytokines

Plasma cytokine concentrations were measured from a separate cohort of control animals and animals 1 day after receiving the same CLP. Blood was centrifuged at 1500×*g* for 10 min at 23 °C, then plasma was stored in a − 80 °C freezer. Concentrations of granulocyte-/macrophage-/granulocyte–macrophage colony-stimulating factor (G-CSF/MCSF/GM-CSF), interleukin (IL)-1α and β, IL-2, IL-4, IL-6, IL-10, IL-12p70, IL-17, tumor necrosis factor (TNF), interferon-γ (IFN-γ), monocyte chemoattractant protein-1 (MCP-1), macrophage inflammatory protein-1α (MIP-1α), and keratinocyte chemoattractant (KC) were determined using the manufacturer’s protocols and using BeadView software (Millipore).

### RNA, Library Preparation, and Sequencing

The transcriptional profile was analyzed in the hippocampus from 12 different groups: young male control (*n* = 4), young male 1 day post-sepsis (*n* = 4), young male 4 days post-sepsis (*n* = 4), old male control (*n* = 4), old male 1 day post-sepsis (*n* = 4), old male 4 days post-sepsis (*n* = 4), young female control (*n* = 4), young female 1 day post-sepsis (*n* = 4), young female 4 days post-sepsis (*n* = 4), old female control (*n* = 4), old female 1 day post-sepsis (*n* = 4), and old female 4 days post-sepsis (*n* = 3). Methods for library preparation and sequencing have previously been published [35, 40]. Briefly, RNA isolation and DNase digestion were performed with RNeasy Lipid Tissue Mini kit (Qiagen, catalog number 74804) and RNase-Free DNase Set (Qiagen, catalog number 79254). The RNA concentration was measured using a NanoDrop 2000 spectrophotometer, and the RNA integrity number (RIN) was quantified on a High Sensitivity RNA ScreenTape in an Agilent 2200 Tapestation system. External RNA Controls Consortium (ERCC) spike-in control (Thermo Fisher, catalog number 4456740) was added to samples as a performance assessment for library preparation. Dynabeads mRNA DIRECT Micro kit (Thermo Fisher, catalog number 61021) selected for poly(A) mRNA and whole transcriptome libraries were prepared with the Ion Total RNA-Seq Kit v2 (Thermo Fisher, catalog number 4475936). Ion Xpress barcodes (Thermo Fisher, catalog number 4475485) were added for multiplex sequencing. Qubit dsDNA High Sensitivity assay (Thermo Fisher, catalog number 32851) and High Sensitivity D1000 ScreenTape in a Tapestation system quantified the concentration and size distribution of the whole transcriptome library. Template preparation was performed using an Ion Chef system and sequenced on an Ion Proton. Analysis of the ERCC control was performed in the Torrent Server with the ERCC analysis plugin. ERCC spiked samples had an *R*^2^ of above 0.9. Each sample had an average of 34 million reads and 148 base pair length. The data for this study has been uploaded to NCBI’s Gene Expression Omnibus under the accession number: GSE128925.

### Bioinformatics and Statistical Analysis

Data analysis for gene expression was performed on the Partek Flow server as previously described [35, 40]. FASTQ files were trimmed and aligned to the mouse (mm10) genome using STAR. Gene counts were normalized using the trimmed mean of M-values (TMM) method [41], and genes with an average total count of less than 5 were removed, consistent with our previously published work [35, 40, 42, 43]. For statistical analysis, counts were log transformed and one-way analyses of variance (ANOVAs) were conducted for the specific analyses described below. For each analysis, the *p* value was set at *p* < 0.01 for statistical filtering. Genes that passed the statistical filter were then separated into upregulated or downregulated based on fold change. These gene lists were then separately submitted to the NIH Database for Annotation, Visualization, and Integrated Discovery (DAVID) for gene enrichment and functional annotation clustering analysis. This analysis was limited to biological processes and cellular components in the “Direct” and “FAT” categories with a Benjamini false discovery rate (FDR) set at *p* < 0.05 as a cutoff for a significant cluster. The significant clusters are reported along with the number of genes in the cluster.

**Fig. 1:**
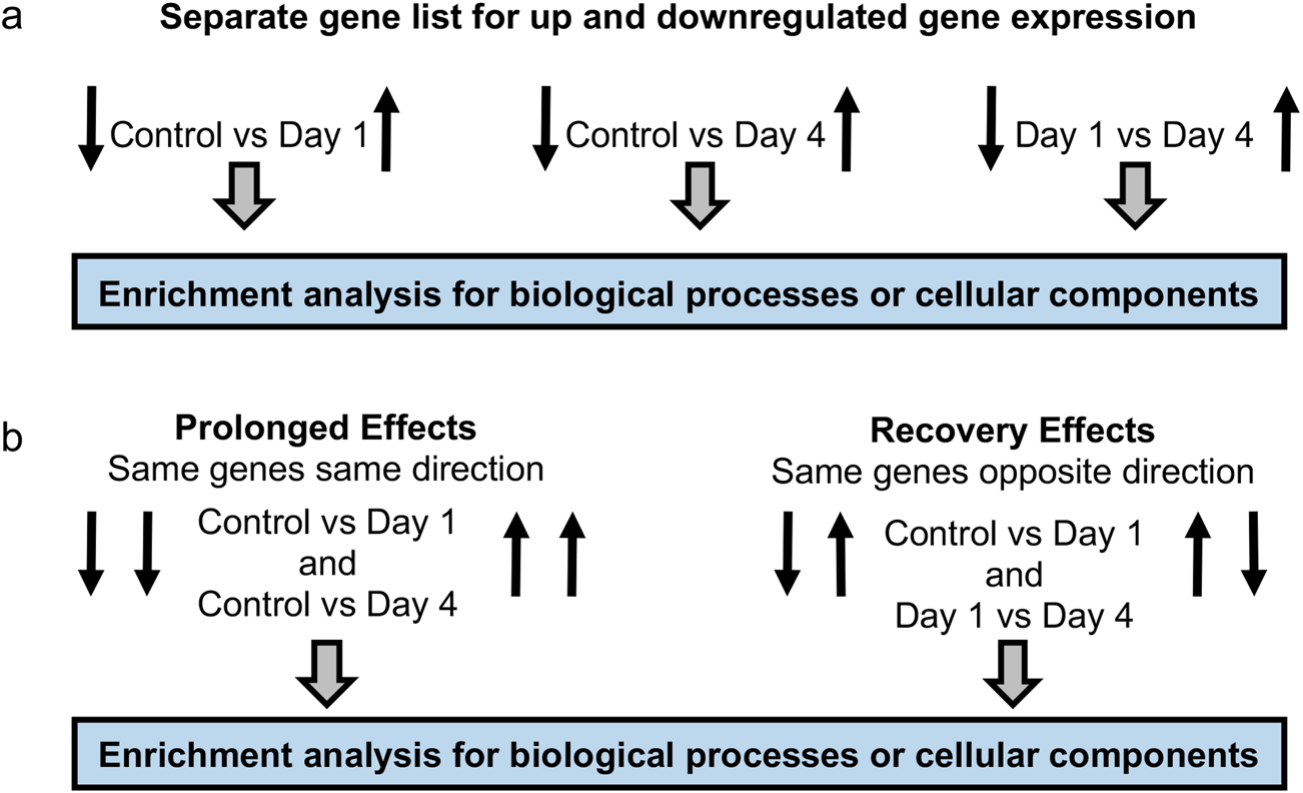
Diagram summarizing the initial analysis performed on the transcriptome. **a** For each age and sex group, gene changes following sepsis were examined by generating lists of differentially expressed genes. Gene lists were generated by statistical filtering (*p* < 0.01), comparing the transcriptome on day 1 or day 4 following sepsis relative to control. Moreover, we examined the changes in expression from day 1 to day 4. Upregulated and downregulated genes were separately analyzed for enrichment of biological processes or cellular components using DAVID. **b** The gene lists generated from the above analysis were then used to examine the prolonged effects or recovery after sepsis. Analysis for the prolonged effect of sepsis was determined by genes that continued to be modified in the same direction on day 1 and day 4 relative to control. For recovery, genes were selected if they were differentially expressed on day 1 (e.g., increasing on day 1) and expression was in the opposite direction on day 4 compared to day 1 (e.g., decreased on day 4 relative to day 1). For prolonged and recovery effects, gene lists were separately analyzed for enrichment of biological processes or cellular components using DAVID

The specific analyses are illustrated in Fig. 1. The response, recovery, and prolonged effects of sepsis were conducted separately within each age and sex. First, the response to sepsis over time was analyzed using one-way ANOVAs between day 1 to control and day 4 to control. Next, we analyzed either the recovery or prolonged effect of sepsis. Analysis to determine if genes exhibited a prolonged dysregulation following sepsis was performed by filtering genes that were either increased or decreased across both time points. Recovery after sepsis was assessed by determining if filtered genes that were initially downregulated (day 1 versus controls) were then upregulated on day 4 compared to day 1. This was also conducted for genes that were initially upregulated (day 1) then downregulated on day 4. The effect of age was analyzed by comparing young and old control animals separately for each sex.

For plasma cytokine analysis, cytokine concentrations were log transformed. Three-way ANOVAs were performed for the factors sepsis, age, and sex. For post hoc analyses, Bonferroni correction was used (*p* < 0.05).

## Results

**Fig. 2:**
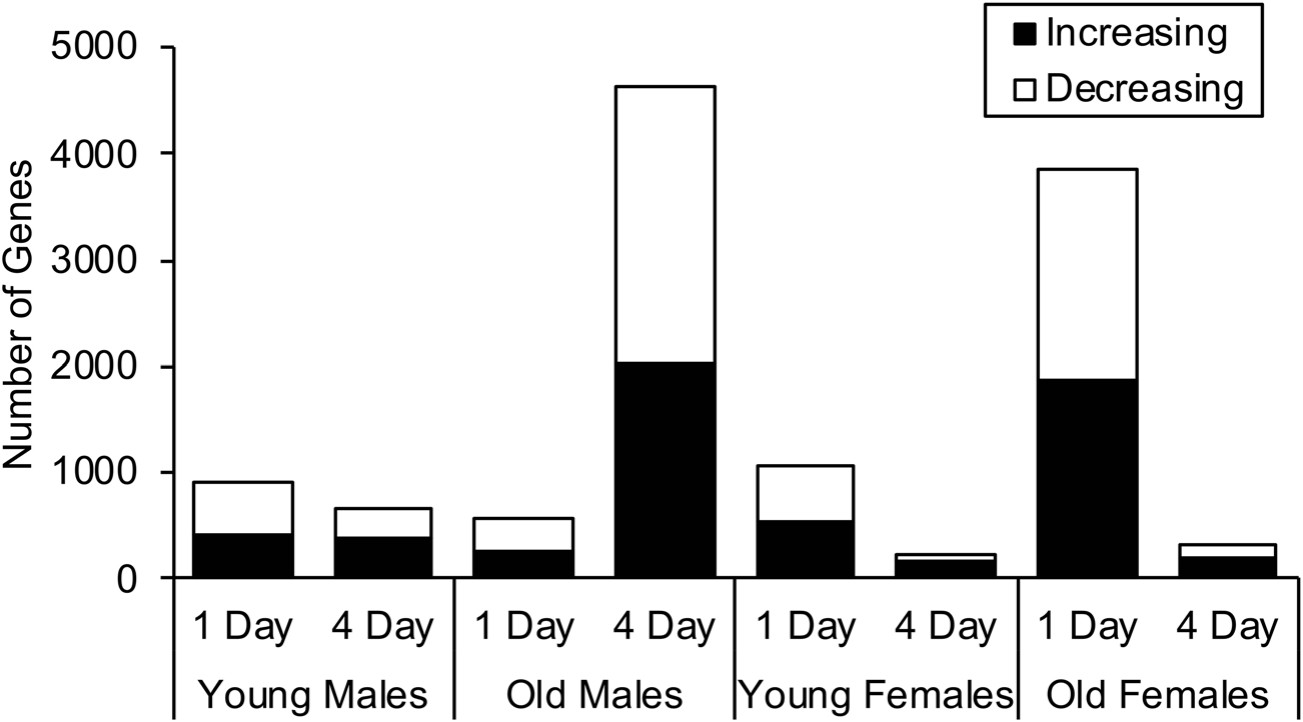
The number of genes differentially expressed for each age and sex group. Summary of the total number of genes that increased (black) or decreased (white) the expression in the hippocampus 1 or 4 days after sepsis relative to age-matched controls

Figure 2 illustrates the number of differentially expressed genes (DEGs) either 1 or 4 days after sepsis relative to age- and sex-matched controls. These genes were further divided based on direction of change (i.e., upregulation or downregulation). Of particular note is that young males, young females, and old females differentially expressed a greater number of genes on day 1 compared to day 4, suggesting recovery over the 4 days. This was particularly evident in old females, which exhibited expression differences fourfold greater than young females on day 1. In contrast, old males exhibited the smallest number of DEGs on day 1, while robust changes were observed on day 4, suggesting a delayed response and/or diminished recovery.

Overall, 241 genes were altered in all four groups in the same direction (116 upregulated and 125 downregulated) on day 1. Upregulated genes were linked to glucocorticoid signaling, including genes that have been observed to increase in the brain following glucocorticoid administration across a number of studies (*Arhgef3*, *Arl4d*, *Cdkn1a*, *Ddit4*, *Errfi1*, *Fkbp5*, *Gjb6*, *Klf9*, *Klf15*, *Nfkbia*, *Pdk4*, *Rhou*, *Tsc22d3*, *Sdc4*, *Sesn1*, *Sgk1*, *Sult1a1*, *Wipf3*). Many of these genes are linked to downstream responses including regulation of transcription, apoptosis, and immunosuppressive effects of glucocorticoids [44]. Other significant clusters included increased expression of genes linked to the regulation of cell growth and proliferation, particularly negative regulation of cell growth and proliferation (Table S1). Enrichment analysis of genes that were downregulated across all groups on day 1 indicated clustering of genes for neurogenesis, gliogenesis, myelination, and lipid and sterol biosynthetic processes.

The number of DEGs that were common across all groups was reduced on day 4, such that only six genes (*Cxxc1*, *Epha10*, *Fkbp5*, *Ly6a*, *Plin4*, *Sgk1*) were upregulated and three genes (*Ednrb*, *P2ry12*, *Rasgrp3*) were downregulated. Interestingly, old males exhibited increased expression of 79 genes on day 4, which had changed in the three other groups by day 1. These genes were largely related to apoptotic process and inflammatory response. Thirty-six genes were downregulated on day 4 in old males, which were originally downregulated on day 1 in the other three groups. These genes were linked to gliogenesis and neurogenesis.

### The Response and Recovery of the Hippocampal Transcriptome Following Sepsis for Each Age and Sex Group

#### Young Males

**Fig. 3:**
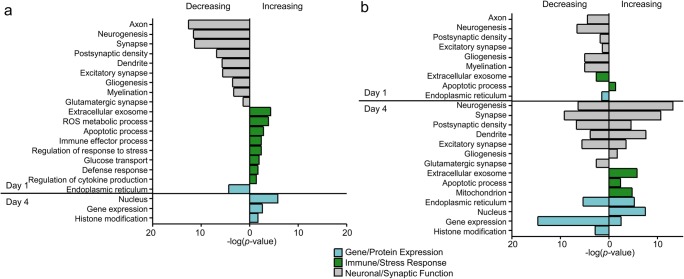
Selected gene ontology (GO) terms for genes that were differentially expressed following sepsis in young (**a**) and old (**b**) males. These clusters were separately generated for genes that were increased or decreased 1 or 4 days after sepsis compared to controls. GO terms were loosely grouped into three main categories: gene/protein expression (blue), immune/stress response (green), and neuronal/synaptic function (gray), and the bar represents the –log(*p* value)

One day after sepsis, young males exhibited a similar number of genes that were upregulated (400) and downregulated (516) compared to controls (Fig. 2). Functional annotation clustering analysis indicated that 1 day after sepsis, genes related to the stress response and neuronal/glial function increased and decreased, respectively (Fig. 3a; Table S2). Genes that were upregulated were related to reactive oxygen species metabolic process, apoptotic process, immune effector response, regulation of response to stress, defense response, regulation of cytokine production, and response to corticosteroid, and positive regulation of phagocytosis was observed. Interestingly, half of the genes for apoptosis were also associated with the gene ontology (GO) category for negative regulation of apoptotic process, although this cluster did not reach significance (*p* = 0.061). Finally, increased expression was observed for genes linked to extracellular exosome and glucose transport.

A robust decrease was observed for genes related to neurogenesis and neuron projection (Fig. 3a; Table S2). Several of the subcategories for the biological process neuron projection were significant (*p* < 0.05), including axon, dendrite, and dendritic spine. In addition, the cellular component synapse was significantly downregulated, including the subcategories postsynaptic density, excitatory synapse, glutamatergic synapse, and *N*-methyl-d-aspartate (NMDA) selective glutamate receptor complex. Further, a significant downregulation was observed for biological processes related to synaptic function, such as synapse organization and synaptic signaling. Other biological processes related to glia were downregulated, including gliogenesis and myelination. Finally, there was a decrease in genes related to the endoplasmic reticulum.

Differential expression analysis comparing controls and 4 days after sepsis indicated fewer DEGs compared to day 1, with increased expression of 386 genes and decreased expression of 268 genes (Fig. 2). It is interesting to note that enrichment analysis indicated no significant clusters for genes that were downregulated 4 days after sepsis. Clustering analysis for genes that increased expression indicated an enrichment for the regulation of transcription, including the nucleus, gene expression, and histone modification (Fig. 3a; Table S2).

**Fig. 4:**
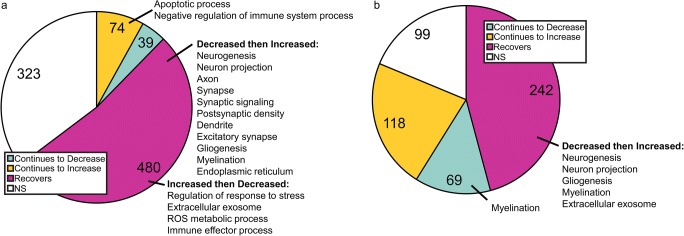
Prolonged dysregulation or recovery of genes that were altered after sepsis in young (**a**) and old (**b**) males. Pie charts illustrate the proportion of genes that either continued to increase (yellow) or decrease (teal) expression across both days compared to controls, genes that recovered expression (pink), and genes that did not exhibit continued dysregulation or recovery across conditions (white; NS = not significant). Recovery was grouped by the direction of change at 1 and 4 days after sepsis. Also, significant clusters associated with each category are listed to the right

Next, the long-term effects of sepsis were examined by comparing genes that were differentially expressed in the same direction 1 and 4 days after sepsis. Very few genes maintained a similar direction of change over the 4 days in young males. A total of 74 genes maintained increased expression and 39 genes continued to exhibit reduced expression (Fig. 4a; Table S2). There were no significant clusters for downregulated genes. Upregulated genes were linked to negative regulation of immune system process and apoptotic process, including 12 genes for the negative regulation of apoptotic process, suggesting that these genes were related to recovery from sepsis.

In addition, we addressed recovery of gene expression that occurred 4 days after sepsis. There were 186 genes that were upregulated on day 1 relative to control and then downregulated on day 4 relative to day 1 (Fig. 4a; Table S2). Functional annotation clustering indicated that these genes were related to regulation of the response to stress, extracellular exosome, reactive oxygen species metabolic process, immune effector process, defense response, and the Kyoto Encyclopedia of Genes and Genomes (KEGG) pathway for the complement and coagulation cascade. A total of 294 genes were initially decreased on day 1 and then increased expression on day 4 relative to day 1 (Fig. 4a; Table S2). Clustering analysis indicated that these genes were related to neurogenesis, neuron projection, axon, synapse, synaptic signaling, postsynaptic density, dendrite, excitatory synapse, gliogenesis, myelination, and endoplasmic reticulum. Thus, 4 days after sepsis, many of the genes related to synaptic function and immune/stress response returned toward normal gene expression levels in young males.

#### Old Males

In contrast to young males, old males appeared to exhibit a delayed response, with most of the transcriptional changes occurring more than 1 day after sepsis. Old males exhibited approximately half the number of DEGs 1 day after infection, compared to young males, with upregulation of 244 genes and downregulation of 311 genes (Fig. 2). Compared to the number of GO terms for young males, old males exhibited a weaker immune/stress response on day 1 with upregulation of genes linked to response to glucocorticoid and apoptotic process (Fig. 3b; Table S3). Similar to young males, genes that were downregulated in aged males were related to the endoplasmic reticulum and to neuronal/synaptic function, including axon, neurogenesis, dendritic spine, postsynaptic density, excitatory synapse, gliogenesis, myelination, and neuron projection. Finally, old males also exhibited decreased expression of genes for extracellular exosome, opposite of that observed in young males.

There were robust changes in the transcriptome 4 days following sepsis, revealing the delayed response in old males. A total of 2039 genes were upregulated and 2586 genes were downregulated relative to controls (Fig. 2). Interestingly, several of the genes linked to glucocorticoid signaling (*Arl4d*, *Cdkn1a*, *Ddit4*, *Fkbp5*, *Gjb6*, *Il6ra*, *Klf15*, *Nfkbia*, *Rhob*, *Sdc4*, *Sgk1*, *Sult1a1*, *Tob2*, *Wipf3*) and apoptotic process were still upregulated 4 days post-sepsis (Fig. 3b; Table S3). Genes related to extracellular exosomes were also upregulated in old males 4 days after sepsis. This cluster was upregulated 1 day after sepsis in young males, consistent with a prolonged or delayed effect of sepsis in old males. Finally, genes linked to mitochondrion were also upregulated 4 days after sepsis, suggesting a possible shift in metabolism.

The observed shift in the old male transcriptomic expression on day 4 may reflect an alteration in the mechanisms that regulate transcription, as old males exhibit a significant upregulation of genes in the nucleus 4 days after sepsis (Fig. 3b; Table S3). Young males exhibited an increased expression of histone modification genes on day 4; however, old males exhibited downregulation of histone-related genes on day 4. This indicates that old males may have a unique transcriptomic response to sepsis relative to younger cohorts. Other clusters for gene regulation exhibited both an up- and downregulation, including gene expression and endoplasmic reticulum.

On day 4, a mixed response was evident in neuronal/glial clusters (Fig. 3b; Table S3). An upregulation and downregulation were observed for genes related to neurogenesis and neuron projection. Subcategories for neuron projection were also differentially expressed, including dendrite and dendritic spine. Furthermore, the cellular compartment synapse was both increased and decreased, including the subcategories postsynaptic density and excitatory synapse. The GO subcategories related to synaptic function, synapse organization, and synaptic signaling were enriched in both upregulated and downregulated genes. Finally, genes specific for the glutamatergic synapse were downregulated 4 days after sepsis. While young males exhibit a marked recovery of synaptic function genes 4 days after sepsis, old males continued to exhibit downregulation of genes related to neuronal/synaptic function.

Finally, we evaluated whether genes continued to display dysregulation across both days or if genes exhibited a recovery of expression. A total of 118 genes increased and 69 decreased expression on day 1 and day 4. Functional enrichment analysis indicated that genes related to myelination continued to exhibit decreased expression 4 days after sepsis (Fig. 4b; Table S3). There were no significant clusters for upregulated genes; however, several genes linked to the immune response (*Fcgr3*, *Jak3*, *Il6ra*, *Il4ra*, *Sh2b2*, *Stat3*, *C1qa*) continued to be elevated. For the 96 genes that exhibited an initial increase and then subsequently decreased expression by day 4 relative to day 1, there were no significant clusters. A total of 173 genes were downregulated 1 day after sepsis but then increased expression on day 4 relative to day 1 (Fig. 4b; Table S3). Clustering analysis indicated that these genes were linked to neurogenesis, neuron projection, gliogenesis, myelination, and extracellular exosome. Thus, while some recovery was noted for genes related to neuronal/synaptic function in old animals, several neuronal/synaptic clusters were still significantly downregulated and stress/immune response genes were upregulated 4 days after sepsis.

#### Young Females

**Fig. 5:**
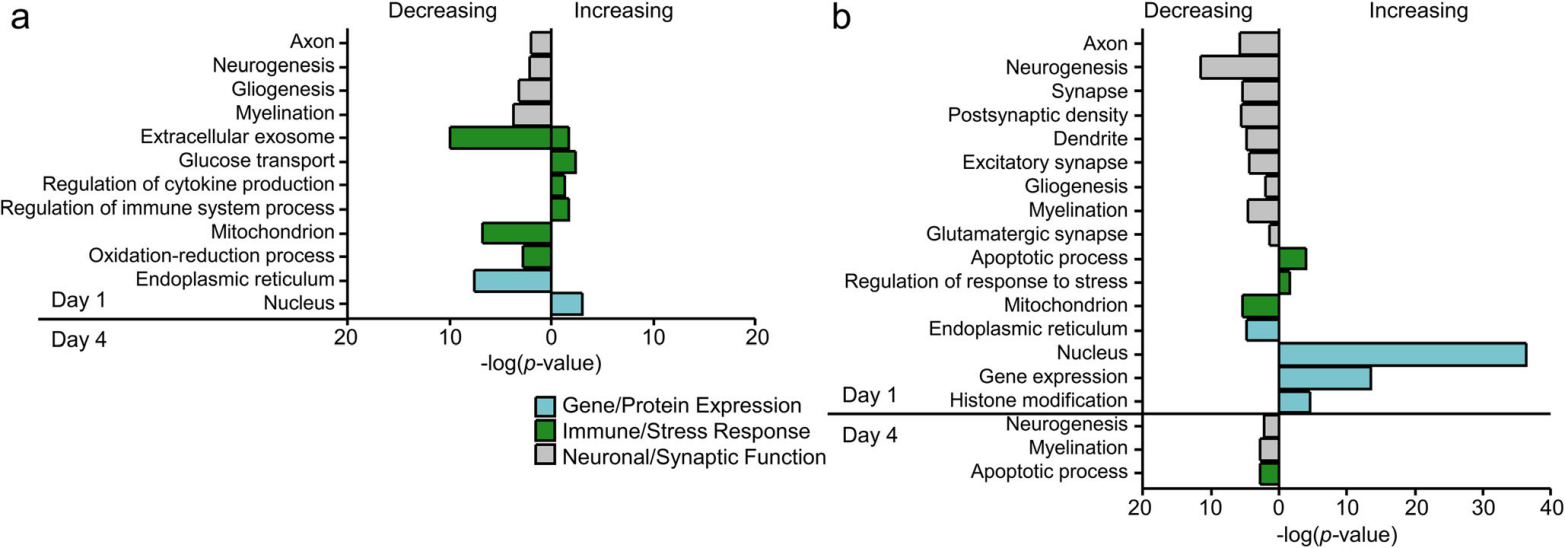
Selected GO terms for genes that were differentially expressed following sepsis in young (**a**) and old females (**b**). Genes that were differentially expressed 1 or 4 days after sepsis were separated into increasing and decreasing. GO terms were loosely grouped into three main categories: gene/protein expression (blue), immune/stress response (green), and neuronal/synaptic function (gray), and the bar represents the –log(*p* value)

One day after sepsis, 524 genes were upregulated and 555 genes were downregulated in the hippocampus of young female mice (Fig. 2). The gene clusters that were modified in young females were similar to those observed in young males, but overall, fewer clusters were observed. Young female mice exhibited increased expression of genes related to stress, including regulation of immune system process and regulation of cytokine production (Fig. 5a; Table S4). Genes linked to oxidation-reduction process were downregulated, while genes related to extracellular exosome were both increased and decreased. Similar to young males, young females increased expression of glucose transport genes. In addition, there was a downregulation of genes related to mitochondrion, indicating a possible alteration of energy metabolism. Unexpectedly, genes related to postsynaptic density were upregulated. However, many other genes related to neuronal and glial function were downregulated, including axon, neurogenesis, gliogenesis, myelination, and neuron projection. Finally, genes related to endoplasmic reticulum were downregulated, similar to that observed in males of both ages. Unlike the males, young females increased expression of genes related to nucleus 1 day after sepsis. This was upregulated in the young males only 4 days after sepsis, suggesting a possible quicker recovery from sepsis in young females.

Relative to the control condition, young females on day 4 exhibited an increased expression of 75 genes and decreased expression of 165 genes (Fig. 2), which is a fewer number of DEGs relative to day 1. No significant clusters were observed for genes that were upregulated post-sepsis. For genes that were downregulated, significant clusters were observed for response to lipid and response to chemical stimulus. The low number of DEGs is consistent with the idea that young females exhibit considerable recovery 4 days after sepsis.

**Fig. 6:**
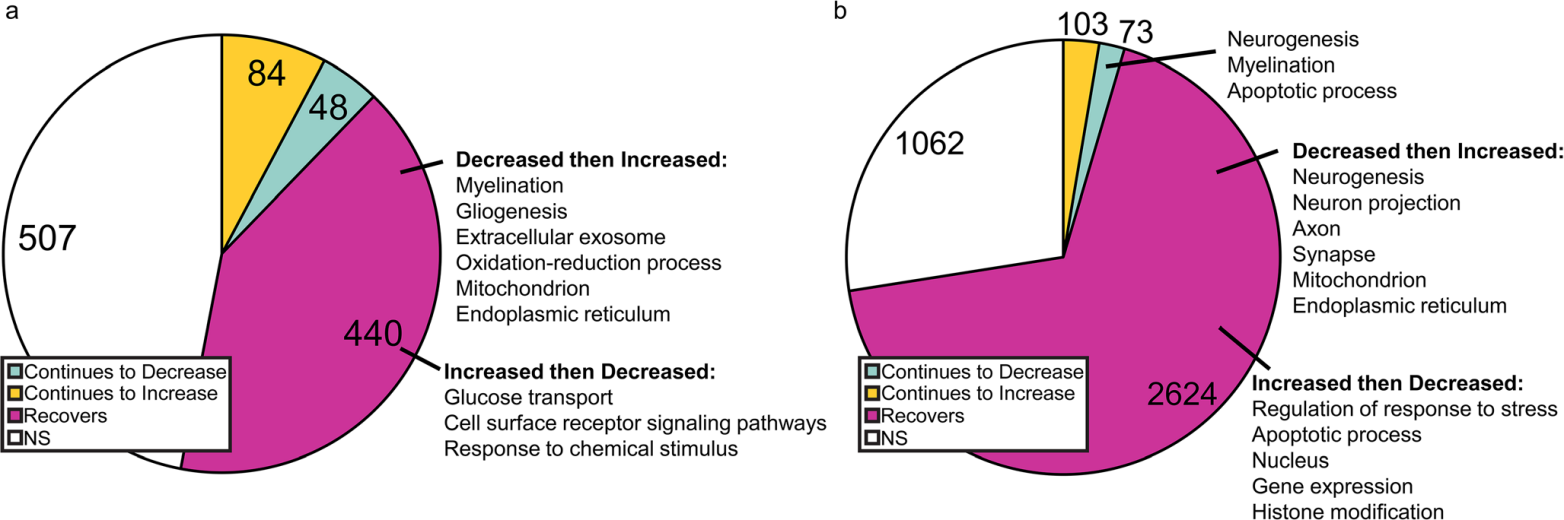
Continued dysregulation or recovery of genes that were altered after sepsis in young (**a**) and old (**b**) females. Pie charts illustrate the proportion of genes that either continued to increase (yellow) or decrease (teal) expression across both days compared to controls, genes that recovered expression (pink), and genes that did not exhibit continued dysregulation or recovery across conditions (white; NS = not significant). Recovery was grouped by the direction of change at 1 and 4 days after sepsis. Also, significant clusters associated with each category are listed to the right

Next, we again assessed continued dysregulation or recovery for genes 4 days after sepsis. Young females displayed very few genes that maintained a similar direction of change over the 4 days. Only 84 genes maintained increased expression and 48 genes that continued to exhibit reduced expression (Fig. 6a; Table S4). There were no significant clusters for genes that continued to be upregulated or downregulated. For genes that recovered over 4 days, a total of 261 genes were downregulated on day 1 and upregulated on day 4 relative to day 1 (Fig. 6a; Table S4). Functional annotation clustering analysis indicated that these genes were related to myelination, gliogenesis, extracellular exosome, oxidation-reduction process, mitochondrion, and endoplasmic reticulum. There were 179 genes that initially increased and subsequently recovered by day 4 (Fig. 6a; Table S4). Enrichment analysis revealed these genes were related to glucose transport, cell surface receptor signaling pathways, and response to chemical stimulus.

Overall, young females exhibited a fewer number of clusters altered at either time point after sepsis compared to other groups. However, the altered clusters were similar to those modified in young males with increasing stress/immune response and decreasing neuronal/glial genes. Neuronal/glial genes that were downregulated in young females recovered 4 days after sepsis, similar to young males. Interestingly, absent from the downregulated genes in young females were clusters related specifically to synaptic function, including synapse, synaptic signaling, and synapse organization.

#### Old Females

One day after sepsis, old females exhibited the largest change in the hippocampal transcriptome with 1859 genes increasing and 2003 genes decreasing compared to controls (Fig. 2). Similar to the young males, old females exhibited an increased expression of genes related to regulation of response to stress (Fig. 5b; Table S5). Moreover, apoptotic process genes were upregulated, similar to that observed in young and old males. However, 52 genes related to the positive regulation of the apoptotic process were downregulated after sepsis, but this cluster did not reach significance. In addition, there was a significant upregulation for genes that regulate gene/protein expression, which included the GO terms nucleus, gene expression, and histone modification. Similar to other groups, genes that were downregulated 1 day after sepsis were related to neuronal/synaptic function, including axon, neurogenesis, synapse, postsynaptic density, dendrite, excitatory synapse, gliogenesis, myelination, glutamatergic synapse, neuron projection, synapse organization, and synaptic signaling. Similar to the other three groups, old females exhibited downregulated expression of genes related to endoplasmic reticulum. Finally, genes related to mitochondrion were observed to be downregulated, suggesting metabolic dysfunction, which was similar to that observed in young females.

Very few genes were differentially expressed 4 days after sepsis in old female mice. Only 194 genes were upregulated and no clusters were observed when functional annotation clustering analysis was performed (Fig. 2). For the 129 genes that were downregulated, clustering analysis was observed for neurogenesis and myelination, indicating a prolonged downregulation of some neuronal/glial genes (Fig. 5b; Table S5). In addition, genes related to apoptotic process were downregulated 4 days after sepsis. This included downregulation of genes in the subcategory positive regulation of apoptotic process, suggesting recovery of apoptotic genes 4 days after sepsis.

The prolonged downregulation of neuronal/glial genes was confirmed through analysis of genes that sustained a similar change from 1 day to day 4 after sepsis. For the 73 genes that were downregulated on day 1 and day 4, functional enrichment analysis indicated clusters related to neurogenesis and myelination (Fig. 6b; Table S5). In addition, genes related to apoptotic process were downregulated across both days, along with the subcategory positive regulation of apoptotic process. There were 100 genes that were upregulated on day 1 and day 4; however, these genes did not exhibit significant clusters (Fig. 6b; Table S5).

For genes that exhibited recovery, 1484 genes decreased on day 1 and increased on day 4 compared to day 1 (Fig. 6b; Table S5). Functional annotation clustering analysis indicated that these genes were related to synaptic function, including neurogenesis, neuron projection, axon, and synapse. Furthermore, other clusters including mitochondrion and endoplasmic reticulum recovered normal expression levels. A total of 1106 genes initially increased transcription on day 1 and then were downregulated on day 4 compared to day 1 (Fig. 6b; Table S5). Clustering analysis indicated that these genes were related to stress response, including the GO terms for the regulation of response to stress and apoptotic process. In addition, recovery was observed for genes that regulate protein expression, including the GO terms nucleus, gene expression, and histone modification. These results indicate that old females are highly responsive to sepsis and exhibit considerable recovery over 4 days. However, genes related to neurogenesis and myelination were still downregulated 4 days after sepsis, indicating a prolonged response or delayed recovery.

### The Influence of the Aging Transcriptome on the Response to Sepsis

**Fig. 7:**
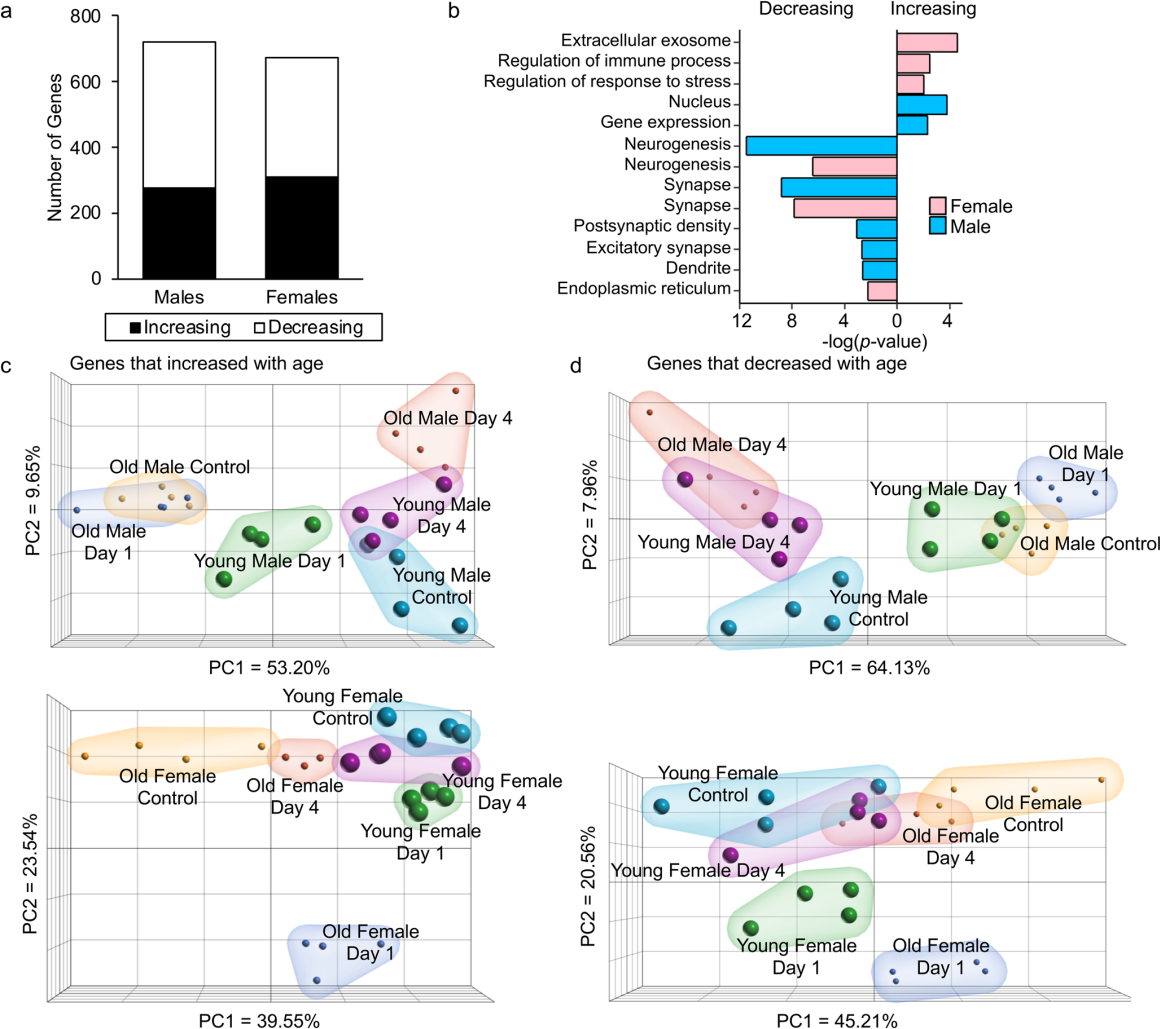
Age-related changes in baseline gene expression and the response to sepsis. **a** The number of DEGs between young and aged control animals separated based on the direction of change and sex. **b** List of selected GO terms that were upregulated or downregulated with age separated based on sex (blue = male; pink = female). The bar represents the –log(*p* value). PCA analysis of the genes that increased (**c**) and decreased (**d**) with age in males (top) and females (bottom)

Many studies have highlighted that the aging hippocampal transcriptome is associated with decreased expression of synaptic genes and increased expression of immune responsive genes [35, 42, 43, 45, 46]. We assessed if the baseline changes in gene expression that occur with age influence the response to sepsis. This was examined by identifying genes that were differentially expressed between young and old controls and then determining if these genes were altered following sepsis. In general, the number of age-related DEGs that were upregulated (male 279; female 309) and downregulated genes (male 444; female 355) was similar in males and females (Fig. 7a). Furthermore, both males and females exhibited enrichment for some of the same GO terms. Similar to previous studies, genes related to neuronal and synaptic genes were downregulated with age. For males, genes that were downregulated with age were enriched for neurogenesis, synapse, postsynaptic density, excitatory synapse, and dendrite (Fig. 7b). Similar to males, aged females exhibit decreased expression of genes related to neurogenesis and synapse. In addition, old females exhibited downregulation of genes related to the endoplasmic reticulum (Fig. 7b).

For females, the increased expression of stress- and immune-linked genes was more evident than in males which included genes linked to extracellular exosome, regulation of immune system process, regulation of response to stress, phagocytosis, and lysosome (Fig. 7b). While clusters related to stress/immune response did not exhibit a significant age difference in males, old males did upregulate several immune genes, including *Ctss*, *Trem2*, *C4b*, and *C3*, which were similarly upregulated in old females. In addition, old females also exhibited an increased expression of several other complement component genes (*C1qa*, *C1qb*, *C1qc*). Finally, enrichment analysis of upregulated genes indicated that males increased expression of genes related to the regulation of transcription, including nucleus and gene expression (Fig. 7b).

**Fig. 8:**
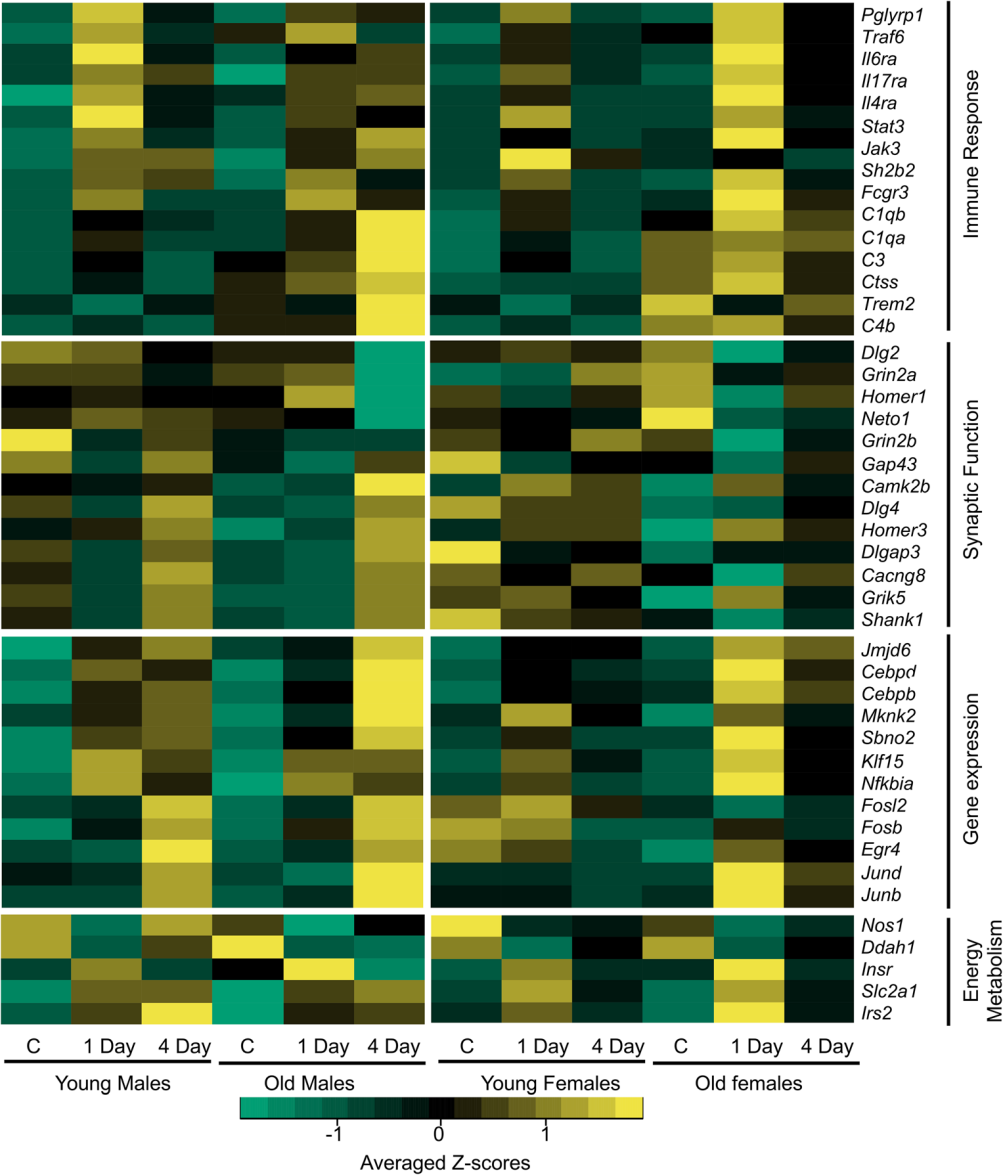
A heatmap illustrating the effect of sepsis on gene expression levels within each age and sex group. Expression levels for each gene were converted into a *z*-score and averaged for each independent group. Genes were grouped into the GO terms immune response, synaptic function, gene expression, and energy metabolism. The color represents the standard deviation, either increasing (yellow) or decreasing (green), relative to the mean (black)

Principal component analyses (PCA) were performed on genes that were upregulated (Fig. 7c) and downregulated (Fig. 7d) with age to visualize how these genes are modified after sepsis. In general, old male mice were unresponsive 1 day after sepsis, revealing a little effect of sepsis on genes that were upregulated or downregulated with age. The unresponsiveness of old males on day 1 could be related to a basal increase in neuroinflammation and decrease in synapse genes with age. As such, gene expression of young males 1 day after sepsis was shifted toward that of old males (Fig. 7c, d). This included increased expression of several genes related to immune function, *Ctss*, *Trem2*, *C4b*, and *C3* (Fig. 8). Finally, 4 days after sepsis, gene expression of young and old males was shifted toward that of young controls, suggesting recovery in the young and a resetting of some aspects of the aging transcriptome for older males. Out of the 444 genes that were downregulated with age, 333 genes were upregulated 4 days after sepsis in old males. Functional enrichment analysis indicated that these genes were related to synapse, dendrite, postsynaptic density, and excitatory synapse (Fig. 8). This resetting of synaptic genes that are downregulated with age contributes to the observation that genes within some neuronal/synaptic categories exhibited up- and downregulation on day 4 in old males (Fig. 3b). Importantly, immune genes that were upregulated with age in the control condition (*Ctss*, *Trem2*, *C4b*) did not exhibit resetting. Rather, these genes exhibited a further increase on day 4 in old males (Fig. 8).

The response of age-related genes in females was considerably different from that in males. First, young females exhibited very little difference in gene expression across treatment conditions. Similar to young males, young females exhibited increased expression of immune and complement component genes *C3*, *C4b*, *C1qa*, and *C1qb* 1 day after sepsis, which returned to baseline by day 4 (Fig. 8). In contrast to old males, genes that were upregulated and downregulated with age were highly responsive on day 1 for old females (Fig. 7c, d). Furthermore, old females exhibited recovery by day 4, with expression halfway between young and old control levels. Finally, while old males exhibited a delayed upregulation of immune genes at day 4, old females exhibited a marked increase in expression of age-related immune and complement component genes on day 1, which recovered on day 4 (Fig. 8).

### Plasma Cytokine Levels Were Altered with Age and Sex and 1 Day After Sepsis

**Fig. 9:**
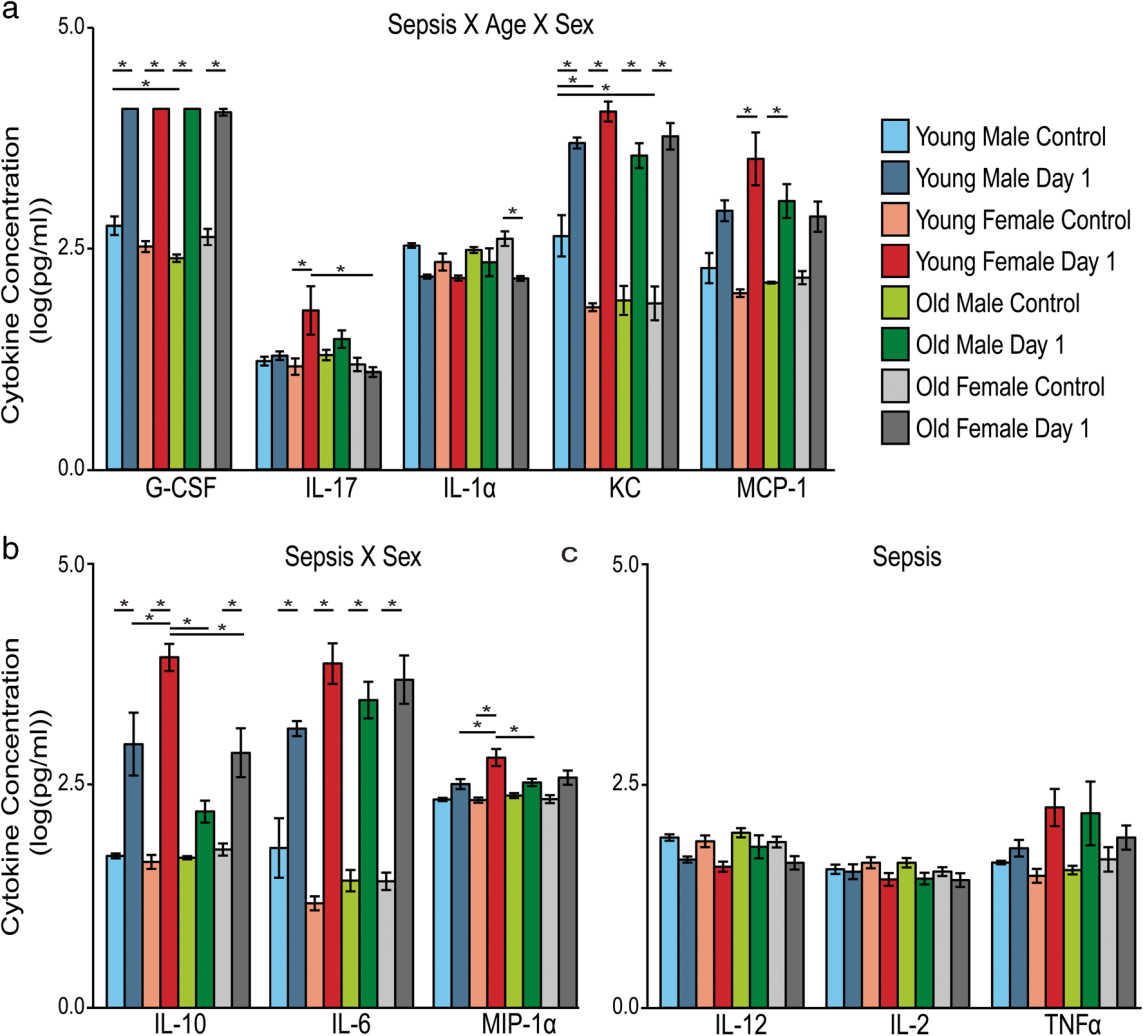
The effect of sepsis, age, and sex on plasma cytokine levels. Bar graphs of the mean cytokine concentration (± SEM) split by group. Cytokines are split into three graphs based on if the cytokine was significant for an interaction or factor **a** sepsis × age × sex interaction, **b** sepsis × sex, and **c** sepsis. *p < 0.05

For the 16 cytokines analyzed, there was a significant effect of sepsis relative to controls for 11 cytokines, with increased levels of [G-CSF (*p* < 0.0001), IL-17(*p* = 0.031), KC (*p* < 0.0001), MCP-1 (*p* < 0.0001), IL-10 (*p* < 0.0001), IL-6 (*p* < 0.0001), MIP-1α (*p* < 0.0001), and TNFα (*p* = 0.001)] and a decrease for IL-1α (*p* < 0.0001), IL-12 (*p* < 0.0001), and IL-2 (*p* = 0.014) (Fig. 9). Five cytokines exhibited a significant sex × sepsis × age interaction [G-CSF (*p* = 0.005), IL-17 (*p* = 0.023), IL-1α (*p* = 0.038), KC (*p* = 0.044), MCP-1 (*p* = 0.0239)] (Fig. 9a). Post hoc analysis indicated an age effect for G-CSF in the control male group where higher cytokine levels were observed in the young compared to the aged (*p* < 0.05). Similarly, IL-17 was elevated in young females compared to aged females 1 day after sepsis (*p* < 0.05). An effect of sex was observed for KC, which was increased in the plasma of control young males compared to control females regardless of age (*p* < 0.05). Several cytokines also exhibited a significant sepsis × sex interaction [IL-6 (*p* = 0.009), IL-10 (*p* = 0.003), MIP-1α (*p* = 0.0149), KC (*p* = 0.003)] (Fig. 9b). This was due to increased levels of MIP-1α and IL-10 in the plasma of young female animals 1 day after sepsis compared to the other groups.

## Discussion

Age is a major risk factor in the outcome of sepsis since the incidence of sepsis and the associated complications increase with advancing age [14–17], including increased risk of neurodegenerative diseases [47]. In addition, sex differences, likely due to sex steroids, underlie the poorer outcome of male sepsis patients [10, 11]. In humans and animal models, sepsis results in long-term impairments in cognition, including memory processes that depend on the hippocampus [4, 48–55]. The mechanisms for long-term cognitive impairments are not clear. Previous research from our laboratory has illustrated that age and sex may induce unique responses to significant inflammation (e.g., severe trauma) [56]. However, this work in sepsis is underinvestigated. While research has focused on the influence of age and sex on mortality and the immune response to sepsis, little research has addressed how age and sex influence the brain’s response to sepsis.

The current study found that a considerable number of hippocampal genes were altered in a similar manner across all sex and age groups on day 1. For example, across all groups, the initial stress following induction of sepsis resulted in upregulation of genes linked to glucocorticoid signaling, including the downstream effects it has on transcriptional regulation [44]. In addition, genes for neurogenesis, gliogenesis, myelination, and lipid and sterol biosynthetic processes were decreased across all groups. In contrast, group differences were observed for the temporal characteristics of gene expression across 4 days. This included genes linked to neural function, stress, and immune response. Thus, a major finding of the current study concerns qualitative differences in the initial responsiveness to sepsis and rate of recovery, associated with age and sex. As discussed below, these gene changes point to possible mechanisms for cognitive impairment and suggest the means by which age and sex interact to determine sepsis outcomes, including long-term cognitive impairment.

Young females were the least responsive group across the 4 days of sepsis, exhibiting the fewest number of altered genes on day 4 and the fewest number of GO clusters (Table 1). When each groups was examined individually on day 1, all groups except young females exhibited an enrichment for an increase in expression of genes linked to apoptosis. The response of young females across the 4 days may contribute to sex-dependent dimorphism in hippocampal recovery after sepsis [57]. The mechanism likely involves increased levels of the anti-inflammatory cytokine, IL-10, and reproductive status [10, 58–60]. Young females may benefit from the effects of estrogen, which is neuroprotective [43, 61–65]. Indeed, the neuroprotective effects of estrogen may be lost as estrogen levels decline with advancing age [62, 66, 67]. Thus, in contrast to young females, older females exhibited a significant enrichment of apoptotic genes on 1 day post-sepsis.

**Table 1:** Direction of change for GO terms that were affected by sepsis in each sex and age group. Each GO term is listed as either increasing, decreasing, or both if it was significantly differentially expressed with age or after sepsis compared to control

GO term/KEGG pathway	Young male		Aged male		Young female		Aged female	
	Day 1	Day 4	Day 1	Day 4	Day 1	Day 4	Day 1	Day 4
Axon	Decrease		Decrease		Decrease		Decrease	
Neurogenesis	Decrease		Decrease	Both	Decrease		Decrease	Decrease
Myelination	Decrease		Decrease		Decrease		Decrease	Decrease
Gliogenesis	Decrease		Decrease	Increase	Decrease		Decrease	
Neuron projection	Decrease		Decrease	Both	Decrease		Decrease	
Postsynaptic density	Decrease		Decrease	Both			Decrease	
Synapse	Decrease			Both			Decrease	
Dendrite	Decrease			Both			Decrease	
Dendritic spine	Decrease			Both				
Glutamatergic synapse	Decrease			Decrease			Decrease	
Endoplasmic reticulum	Decrease	Increase	Decrease	Both	Decrease		Decrease	
Histone modification		Increase		Decrease			Increase	
Gene expression		Increase		Both			Increase	
RNA metabolic process		Increase		Both			Increase	
Regulation of cytokine production	Increase				Increase			
Regulation of response to stress	Increase	Decrease					Increase	
Response to corticosteroid	Increase		Increase					
Apoptotic process	Increase		Increase	Increase			Increase	Decrease
Extracellular exosome	Increase		Decrease	Increase	Decrease			
Glucose transport	Increase				Increase			
Mitochondrion				Increase	Decrease		Decrease	

Older animals exhibited a prolonged transcriptional response or delayed transcriptional recovery to sepsis, particularly older males. The genes that were altered on day 4 in older animals may shed light on the mechanism for sepsis-induced cognitive impairment, which is particularly evident in older individuals [4, 68–70]. Decreased expression for neurogenesis and myelination genes was observed in older males and females on day 4. Inflammation-mediated inhibition of neurogenesis has been linked to impaired cognition [71–73]. In addition, inflammation impairs glutamatergic synaptic plasticity involved in memory function [32, 33, 74]. Older males exhibited a decrease in expression of glutamatergic synaptic genes on day 4. The prolonged activation of apoptotic processes in the hippocampus in older males is consistent with the idea that systemic inflammation can contribute to age-related neurodegenerative diseases [75, 76]. The mechanism for the prolonged response in older males is unclear but may be a consequence of a delayed peripheral immune response [18, 77, 78].

The hippocampus of older animals may be predisposed to respond differently to sepsis due to an age-related increased expression of immune response genes and decreased expression of synaptic genes. For immune response genes, several genes related to the complement cascade were increased with age and further increased over the 4 days after sepsis. Thus, age-related differences in the resiliency or vulnerability to sepsis may depend on the pre-existing neuroinflammatory state. It is interesting that several synaptic genes that were downregulated with age increased on day 4 after sepsis in aged males. The functional significance behind this upregulation of synaptic genes is unclear. The increase may be due to altered behavior and neural activity associated with sepsis. Previous research has described an increased expression of synaptic genes during the progression from normal aging to neurodegenerative disease [79, 80], including genes we observed to increase in older males on day 4 of sepsis (*Cacnb1*, *Cacna1a*, *Ephb4*, *Glul*, *Jph3*, *Mink1*, *Nrxn2*, *Grasp*).

## Conclusions

These results indicate sex and age influence the response of several key biological processes following sepsis, including immune response, neuronal/synaptic function, and regulation of transcription. Aged males exhibit a delayed or prolonged transcriptional response of immune and neuronal/glial genes. Aged females exhibited considerable recovery over 4 days; however, a delay in recovery was observed for genes related to neurogenesis and myelination. Young females exhibited a muted response, suggesting that they are less responsive to the effects of sepsis. The distinctive transcriptional profiles within each age and sex group suggest mechanisms for the resiliency or susceptibility to cognitive decline associated with sepsis. A prolonged decline in glial, neuronal, and synaptic genes may portend the disruption of synaptic connectivity and the extent of cognitive impairment. Similarly, the influence of sepsis on apoptotic processes may contribute to increased susceptibility to neurodegeneration of older individuals.

## Electronic Supplementary Material

ESM 1 (DOCX 26 kb)
