# Supplementary material for: Age and Sex Influence the Hippocampal Response and Recovery Following Sepsis
Source: Mol Neurobiol. 2019 Jul 5;56(12):8557–72. doi: 10.1007/s12035-019-01681-y (PMC6834928; doi:10.1007/s12035-019-01681-y)
Supplement: Supplementary file 1 — (DOCX 26 kb) [file 12035_2019_1681_MOESM1_ESM.docx]

| **Supplementary Table 1:** | | |
| --- | --- | --- |
| GO Term/ KEGG Pathway | *p*-value | Genes |
| Regulation of cell growth | ↑ *p*=7.6E-04 | *Sgk1, Sgk3, Igfbp7, Adipor2, Sesn1, Cdkn1a, Fam107a, Htra1, Agt, Fbln5, Htra3, Apbb2, Ip6k2, Spp1* |
| Negative regulation of cell growth | ↑ p=4.4E-02 | *Cdkn1a, Agt, Adipor2, Sesn1, Apbb2, Ip6k2, Spp1* |
| Regulation of cell proliferation | ↑ p=3.1E-03 | *Xdh, B4galt1, Txnip, Irs2, Icosl, Sgk1, Klf9, Sgk3, Osmr, Acer2, Il4ra, Nfkbia, Gjb6, Sdc4, Stat3, Il6ra, Tns2, Cdkn1a, Apod, Ptgds, Htra1, Agt, Idh2, Tgm2, Errfi1, Ccar1* |
| Negative regulation of cell proliferation | ↑ p=7.6E-02 | *Xdh, B4galt1, Cdkn1a, Tns2, Ptgds, Apod, Klf9, Agt, Idh2, Gjb6, Sdc4, Stat3* |
| Response to hormone | ↑ p=1.5E-04 | *Txnip, Irs2, Sgk1, Klf9, Igfbp7, Pdk4, Adipor2, Timp4, Stxbp3, Stat3, Ddit4, Serpina3n, Cdkn1a, Ptgds, Ucp2, Agt, Sult1a1, Sh2b2, Kdm3a, Errfi1, Spp1* |
| Response to glucocorticoid | ↑ p=1.3E-02 | *Cdkn1a, Sgk1, Ptgds, Klf9, Sult1a1, Igfbp7, Errfi1, Ddit4* |
| Apoptotic process | ↑ p=1.2E-02 | *Xdh, Ier3, Pglyrp1, Tsc22d3, Agt, Tgm2, Ip6k2, Ccar1, Spp1, B4galt1, Txnip, Irs2, Sgk1, Sgk3, AcerR2, Pdk4, Gjb6, Stat3, Ddit4, Dapk1, Cdkn1a, Ucp2, Hif3a, Apbb2, Cbs* |
| Glucose transport | ↑ p=1.2E-02 | *Irs2, Agt, Slc2a1, Adipor2, Stxbp3, Sh2b2, Klf15* |
| Lipid biosynthesis | ↓ p=1.8E-07 | *Msmo1, Scd2, Fa2h, Dhcr7, Fdps, Hmgcs1, Fads2, Acly, Mid1ip1, Idi1, Dhcr24, Nsdhl* |
| Sterol biosynthesis | ↓ p=2.5E-07 | *Msmo1, Dhcr7, Fdps, Hmgcs1, Idi1, Dhcr24, Nsdhl* |
| Gliogenesis | ↓ p=1.9E-03 | *P2ry12, Mag, Plp1, Fa2h, Phgdh, Ptn, Ccp, Il33, Lpar1, Plpp3, Gap43* |
| Myelination | ↓ p=8.3E-03 | *Mag, Plp1, Ugt8A, Fa2h, Pllp, Lpar1, Gal3st1* |
| Neurogenesis | ↓ p=8.8E-04 | *Fgfr2, Mag, Cav1, Plp1, Nos1, Myo6, Tubb2b, Ugt8a, Fa2h, Plxnb3, Rorb, Cnp, Lpar1, Il33, Gprc5b, Ephb1, Plpp3, Kdr, P2ry12, Ednrb, Unc5b, Mapt, Phgdh, Ptn, Ncan, Lrp4, Gap43* |

**Table 1** A list of the significant GO terms that were upregulated or downregulated across all age and sex groups 1 day after sepsis. The *p*-value and genes associated with that GO term are provided.

**Table 2** Table depicting how certain GO terms are affected in young males based on the different analysis performed. The direction of change (↑ increased; ↓ decreased), *p*-value, the number of genes associated with that GO term are provided.

| **Supplementary Table 2:** | | | | | | |
| --- | --- | --- | --- | --- | --- | --- |
| GO Term/ KEGG Pathway | Acute v Control | Chronic v Control | Prolonged Increase | Recovery (Increase to Decrease) | Prolonged Decrease | Recovery (Decrease to Increase) |
| ROS metabolic process | ↑ *p*=1.63E-04;  21 genes |  |  | *p*=3.82E-02;  10 genes |  |  |
| Apoptotic process | ↑ *p*=1.67E-03;  65 genes |  | *p*=3.0E-02;  20 genes |  |  |  |
| Immune effector process | ↑ *p*=4.20E-03;  34 genes |  |  | *p*=3.13E-02;  19 genes |  |  |
| Regulation of response to stress | ↑ *p*=4.23E-03;  46 genes |  |  | *p*=2.0E-02;  26 genes |  |  |
| Defense response | ↑ *p*=1.92E-02;  50 genes |  |  | *p*=3.07E-02;  29 genes |  |  |
| Regulation of cytokine production | ↑ *p*=4.39E-02;  24 genes |  |  |  |  |  |
| Response to glucocorticoid | ↑ *p*=3.23E-02;  12 genes |  |  |  |  |  |
| Positive regulation of phagocytosis | ↑ *p*=4.65E-02;  7 genes |  |  |  |  |  |
| Extracellular exosome | ↑ *p*=4.92E-05;  88 genes |  |  | *p*=2.1E-04;  48 genes |  |  |
| Glucose Transport | ↑ *p*=1.39E-02;  11 genes |  |  |  |  |  |
| Neurogenesis | ↓ *p*=2.46E-12;  97 genes |  |  |  |  | *p*=6.41E-07;  57 genes |
| Neuron projection | ↓ *p*=6.17E-19;  101 genes |  |  |  |  | *p*=8.61E-13;  63 genes |
| Axon | ↓ *p*=2.19E-13;  42 genes |  |  |  |  | *p*=2.41E-08;  26 genes |
| Dendrite | ↓ *p*=2.03E-06;  46 genes |  |  |  |  | *p*=1.43E-05;  25 genes |
| Dendritic spine | ↓ *p*=4.22E-02;  11 genes |  |  |  |  |  |
| Synapse | ↓ *p*=3.64E-12;  70 genes |  |  |  |  | *p*=2.01E-06;  40 genes |
| Postsynaptic density | ↓ *p*=1.29E-07;  26 genes |  |  |  |  | *p*=5.04E-04;  15 genes |
| Excitatory synapse | ↓ *p*=2.31E-06;  27 genes |  |  |  |  | *p*=3.75E-03;  15 genes |
| Glutamatergic synapse | ↓ *p*=4.16E-02;  219 genes |  |  |  |  |  |
| NMDA selective gluatamate receptor complex | ↓ *p*=3.52E-02;  4 genes |  |  |  |  |  |
| Synapse organization | ↓ *p*=2.74E-08;  28 genes |  |  |  |  |  |
| Synaptic signaling | ↓ *p*=1.10E-07;  43 genes |  |  |  |  | *p*=9.2E-04;  24 genes |
| Gliogenesis | ↓ *p*=2.53E-04;  23 genes |  |  |  |  | *p*=4.03E-02;  13 genes |
| Myelination | ↓ *p*=4.59E-04;  14 genes |  |  |  |  | *p*=3.76E-03;  10 genes |
| Endoplasmic reticulum | ↓ *p*=4.13E-05;  63 genes |  |  |  |  | *p*=1.41E-02;  35 genes |
| Nucleus |  | ↑ *p*=1.54E-06;  157 genes |  |  |  |  |
| Gene expression |  | ↑ *p*=2.44E-03;  137 genes |  |  |  |  |
| Histone modification |  | ↑ *p*=2.33E-02;  20 genes |  |  |  |  |
| Negative regulation of the immune system |  |  | *p*=3.3E-02;  9 genes |  |  |  |
| Complement and coagulation cascade |  |  |  | *p*=4.26E-02;  6 genes |  |  |

**Table 3** Table depicting how certain GO terms are affected in old males based on the different analysis performed. The direction of change (↑ increased; ↓ decreased), *p*-value, the number of genes associated with that GO term are provided.

| **Supplementary Table 3: Aged males** | | | | | | |
| --- | --- | --- | --- | --- | --- | --- |
| GO Term/ KEGG Pathway | Acute v Control | Chronic v Control | Prolonged Increase | Recovery (Increase to Decrease) | Prolonged Decrease | Recovery (Decrease to Increase) |
| Response to glucocorticoid | ↑ *p*=1.63E-02;  11 genes |  |  |  |  |  |
| Apoptotic process | ↑ *p*=4.47E-02;  39 genes | ↑ *p*=4.48E-03; 221 genes |  |  |  |  |
| Endoplasmic reticulum | ↓ *p*=2.82E-02;  34 genes | ↑ *p*=7.17E-06; 184 genes ↓ p=4.87E-06; 222 genes |  |  |  |  |
| Axon | ↓ *p*=3.06E-05;  21 genes |  |  |  |  |  |
| Neurogenesis | ↓ *p*=2.44E-07;  60 genes | ↑ *p*=7.52E-14; 264 genes ↓ p=4.42E-07; 275 genes |  |  |  | *p*=1.21E-05; 38 genes |
| Dendritic spine | ↓ *p*=9.43E-06;  14 genes | ↑ *p*=1.24E-04; 34 genes ↓ p=1.31E-04; 39 genes |  |  |  |  |
| Postsynaptic density | ↓ *p*=1.51E-02; 12 genes | ↑ *p*=2.81E-05; 49 genes ↓ p=1.68E-07; 63 genes |  |  |  |  |
| Excitatory synapse | ↓ *p*=4.58E-02; 13 genes | ↑ *p*=3.39E-04; 54 genes ↓ p=2.50E-06; 69 genes |  |  |  |  |
| Gliogenesis | ↓ *p*=4.58E-02; 13 genes | ↑ *p*=2.34E-02; 46 genes |  |  |  | *p*=2.98E-06; 17 genes |
| Myelination | ↓ *p*=9.30E-06; 21 genes |  |  |  | *p*=2.3E-02; 5 genes | *p*=2.13E-04; 10 genes |
| Neuron projection | ↓ *p*=3.38E-03; 44 genes | ↑ *p*=4.10E-11; 220 genes ↓ p=2.25E-08; 246 genes |  |  |  | *p*=1.17E-02; 28 genes |
| Extracellular exosome | ↓ *p*=2.28E-03; 64 genes | ↑ *p*=1.75E-06; 337 genes |  |  |  | *p*=1.99E-03; 41 genes |
| Mitochondrion |  | ↑ *p*=2.04E-05; 226 genes |  |  |  |  |
| Nucleus |  | ↑ *p*=3.45E-08; 696 genes |  |  |  |  |
| Histone modification |  | ↓ *p*=1.43E-03; 79 genes |  |  |  |  |
| Gene expression |  | ↑ *p*=3.26E-03; 583 genes ↓ p=2.12E-15; 804 genes |  |  |  |  |
| Dendrite |  | ↑ *p*=2.63E-08; 123 genes ↓ p=1.43E-04; 94 genes |  |  |  |  |
| Synapse |  | ↑ *p*=2.12E-11; 169 genes ↓ p=5.90E-10; 192 genes |  |  |  |  |
| Synapse organization |  | ↑ *p*=3.54E-06; 53 genes ↓ p=4.93E-03; 50 genes |  |  |  |  |
| Synaptic signaling |  | ↑ *p*=8.31E-03; 83 genes ↓ p=9.92E-03; 98 genes |  |  |  |  |
| Glutamatergic synapse |  | ↓ *p*=2.25E-03; 28 genes |  |  |  |  |

**Table 4** Table depicting how certain GO terms are affected in young females based on the different analysis performed. The direction of change (↑ increased; ↓ decreased), *p*-value, the number of genes associated with that GO term are provided.

| **Supplementary Table 4: Young female** | | | | | | |
| --- | --- | --- | --- | --- | --- | --- |
| GO Term/ KEGG Pathway | Acute v Control | Chronic v Control | Prolonged Increase | Recovery (Increase to Decrease) | Prolonged Decrease | Recovery (Decrease to Increase) |
| Regulation of immune system process | ↑ p=2.33E-02; 52 genes |  |  |  |  |  |
| Regulation of cytokine production | ↑ p=4.6E-02; 28 genes |  |  |  |  |  |
| Oxidation-reduction process | ↓ p=1.64E-03; 49 genes |  |  |  |  | p=6.9E-03; 23 genes |
| Extracellular exosome | ↑ p=1.96E-02; 93 genes ↓ p=1.04E-10; 130 genes |  |  |  |  | p=7.44E-06; 63 genes |
| Glucose Transport | ↑ p=4.48E-03; 13 genes |  |  |  |  | p=1.56E-02; 8 genes |
| Mitochondrion | ↓ p=1.71E-07; 87 genes |  |  |  |  | p=1.88E-06; 49 genes |
| Postsynaptic density | ↑ p=2.01E-02; 17 genes |  |  |  |  |  |
| Axon | ↓ p=8.82E-03; 23 genes |  |  |  |  |  |
| Neurogenesis | ↓ p=6.46E-03; 71 genes |  |  |  |  |  |
| Gliogenesis | ↓ p=6.36E-04; 24 genes |  |  |  |  | p=1.14E-03; 16 genes |
| Myelination | ↓ p=1.81E-04; 16 genes |  |  |  |  | p=3.73E-05; 13 genes |
| Neuron projection | ↓ p=4.66E-03; 64 genes |  |  |  |  |  |
| Endoplasmic reticulum | ↓ p=2.55E-08; 75 genes |  |  |  |  | p=1.46E-08; 47 genes |
| Nucleus | ↑ p=8.68E-04; 193 genes |  |  |  |  |  |
| Cell surface receptor signaling pathways |  |  |  |  |  | p=5.19E-03; 41 genes |
| Response to chemical stimulus |  |  |  |  |  | p=6.59E-03; 45 genes |

**Table 5** Table depicting how certain GO terms are affected in old females based on the different analysis performed. The direction of change (↑ increased; ↓ decreased), *p*-value, the number of genes associated with that GO term are provided.

| **Supplementary Table 5: Aged female** | | | | | | |
| --- | --- | --- | --- | --- | --- | --- |
| GO Term/ KEGG Pathway | Acute v Control | Chronic v Control | Prolonged Increase | Recovery (Increase to Decrease) | Prolonged Decrease | Recovery (Decrease to Increase) |
| Regulation of response to stress | ↑ *p*=3.07E-02; 138 genes |  |  | *p*=5.81E-03; 96 genes |  |  |
| Apoptotic process | ↑ *p*=1.25E-04; 89 genes | ↓ *p*=1.79E-03; 29 genes |  | *p*=7.6E-04; 60 genes | *p*=2.25E-02; 17 genes |  |
| Nucleus | ↑ *p*=3.99E-37; 770 genes |  |  | *p*=3.85E-21; 462 genes |  |  |
| Gene expression | ↑ *p*=3.61E-14; 613 genes |  |  | *p*=1.37E-06; 367 genes |  |  |
| Histone modification | ↑ *p*=2.14E-05; 70 genes |  |  | *p*=1.15E-04; 48 genes |  |  |
| Axon | ↓ *p*=1.76E-06; 70 genes |  |  |  |  | *p*=2.69E-04; 51 genes |
| Neurogenesis | ↓ *p*=3.18E-12; 249 genes | ↓ *p*=5.99E-03; 25 genes |  |  | *p*=5.46E-04; 20 genes | *p*=2.23E-02; 151 genes |
| Synapse | ↓ *p*=4.40E-06; 142 genes |  |  |  |  | *p*=2.00E-02; 56 genes |
| Postsynaptic density | ↓ *p*=3.56E-06; 51 genes |  |  |  |  |  |
| Dendrite | ↓ *p*=2.08E-05; 81 genes |  |  |  |  |  |
| Excitatory synapse | ↓ *p*=5.36E-05; 55 genes |  |  |  |  |  |
| Gliogenesis | ↓ *p*=1.29E-02; 47 genes |  |  |  |  |  |
| Myelination | ↓ *p*=2.54E-05; 31 genes | ↓ *p*=1.62E-03; 8 genes |  |  | *p*=2.13E-02; 5 genes |  |
| Glutamatergic synapse | ↓ *p*=3.44E-02; 23 genes |  |  |  |  |  |
| Neuron projection | ↓ *p*=7.57E-07; 197 genes |  |  |  |  | *p*=1.57E-02; 133 genes |
| Synapse organization | ↓ *p*=2.14E-02; 40 genes |  |  |  |  |  |
| Synaptic signaling | ↓ *p*=1.99E-05; 93 genes |  |  |  |  |  |
| Endoplasmic reticulum | ↓ *p*=2.09E-05; 177 genes |  |  |  |  | *p*=2.05E=-03; 128 genes |
| Mitochondrion | ↓ *p*=4.00E-06; 225 genes |  |  |  |  | *p*=2.0E-08; 186 genes |
| Positive regulation of apoptotic process |  |  |  |  | *p*=4.25E-02; 9 genes |  |

**Table 6** A list of the significant GO terms that were altered with age. The direction of change (↑ increased; ↓ decreased), *p*-value, the number of genes associated with that GO term are provided.

| **Supplementary Table 6:** | | |
| --- | --- | --- |
| GO Term/ KEGG Pathway | Male | Female |
| Neurogenesis | *↓ p*=3.42E-12; 86 genes | *↓ p*=3.77E-07; 65 genes |
| Synapse | *↓ p*=1.58-09; 39 genes | *↓ p*=1.36E-08; 33 genes |
| Postsynaptic density | *↓ p*=8.46-04; 18 genes |  |
| Excitatory synapse | *↓ p*=2.07E-03; 19 genes |  |
| Dendrite | *↓ p*=2.36E-03; 26 genes |  |
| Endoplasmic reticulum |  | *↓ p*=6.01E-03; 42 genes |
| Extracellular exosome |  | *↑ p*=2.52E-05; 73 genes |
| Regulation of immune system process |  | *↑ p*=3.34E-03; 41 genes |
| Regulation of response to stress |  | *↑ p*=8.86E-03; 38 genes |
| Phagocytosis |  | *↑ p*=3.25E-03; 14 genes |
| Lysosome |  | *↑ p*=7.87E-04; 18 genes |
| Nucleus | *↑ p*=1.69E-04; 112 genes |  |
| Gene expression | *↑ p*=4.76-03; 97 genes |  |
